# Exploring the divergence of rare earth trade networks with a global simulation model

**DOI:** 10.1016/j.isci.2025.113658

**Published:** 2025-09-27

**Authors:** Yawen Han, Peng Wang, Zhongju Liao, Linbin Tang, Wenjuan Song, Tianming Gao, Hongchang Hao, Wei-qiang Chen

**Affiliations:** 1 School of Economics and Management, Zhejiang Sci-Tech University, No. 928, 2nd Street, Qiantang District, Hangzhou 310018, China; 2 State Key Laboratory for Ecological Security of Regions and Cities, Institute of Urban Environment, Chinese Academy of Sciences, No.1799 Jimei Road, Jimei District, Xiamen 361021, China; 3 Research Center for Strategy of Global Mineral Resources, Chinese Academy of Geological Sciences, No. 26 Baiwanzhuang Street, Xicheng District, Beijing 100037, China; 4 State Key Laboratory of Regional and Urban Ecology, Institute of Urban Environment, Chinese Academy of Sciences, No.1799 Jimei Road, Jimei District, Xiamen 361021, China; 5 University of Chinese Academy of Sciences, No.1 Yanqihu East Road, Huairou District, Beijing 100049, China

**Keywords:** Earth sciences, Natural resources, Energy policy, Political science

## Abstract

Rare earths are critical to high-tech and low-carbon applications. To protect national interests, countries increasingly adopt geopolitical trade actions among rare earth supply chains. Here, this study combines a global simulation model with network analysis to reveal how trade barriers, coalitions, and liberalization could reshape international rare earth trade networks. Results show an increasingly reduced concentration of global trade patterns, which have divided into more regional groups. In particular, Japan, Malaysia, and several Asian countries have formed a community increasingly mediating China–U.S. trade. Scenario analysis highlights the different role of actions from major exporters in shaping global networks, and trade barriers would intensify regionalization tendency, while non-discriminatory coalitions and trade liberalization can encourage globalization. Our analysis underscores the necessity of broader cooperation for a resilient rare earth supply chain.

## Highlights

• Dynamics of international rare earth trade under trade uncertainty are explored
• Emerging exporters can have a notable impact on global rare earth supply
• Trade barriers will reinforce the regionalization of the rare earth trade network
• Trade liberalization can slightly foster globalization in the rare earth trade

## Graphical abstract

**Figure:**
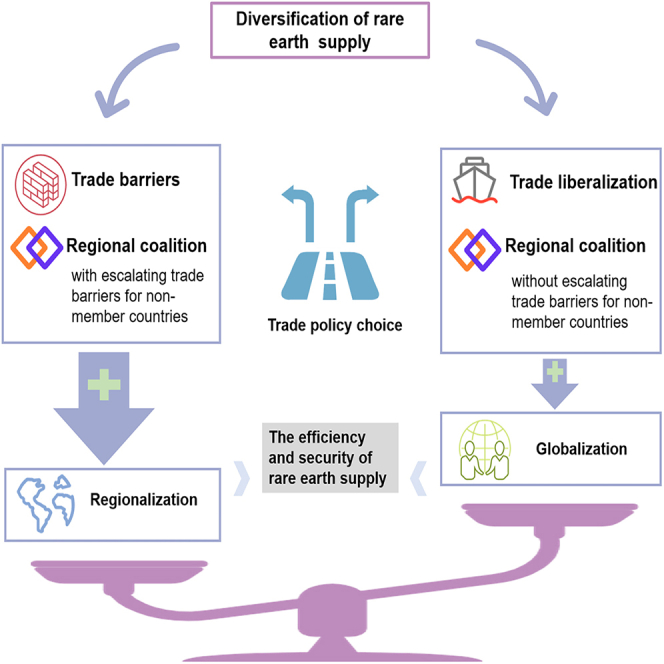

## Introduction

Rare earths are widely recognized as critical minerals essential to modern economies.^1^ From a supply perspective, rare earth deposits are highly unevenly distributed across the globe, with China, Brazil, India and Australia collectively holding over 86% of the world’s total known reserves.^2^ Moreover, rare earth mine production is led by China and the United States (the U.S.), which together contribute more than 80% of total output worldwide.^2^ In contrast, certain high-end applications and advanced technological patents are held by the U.S. and Japan.^3^ These imbalances in resource availability, production capacity, and technological advancement makes international trade as the key factor in redistributing rare earth resources to meet diverse regional demands. From the demand perspective, climate targets are expected to drive a significant surge in the need for rare earths. For example, the demand for dysprosium and neodymium for clean energy applications is projected to increase by more than 5-fold by 2050 compared with 2023.^4^ This sharp rise in demand poses various risks such as inadequate production capacity and potential trade disruptions. Currently, the growing geopolitical tensions and trade protectionism further complicate the situation.^5^^,^^6^ Some countries tend to impose trade barriers to safeguard their national interests, while regional trade coalitions risk becoming increasingly discriminatory.^7^ For example, the Mineral Security Partnership (MSP), comprising 14 countries and the European Union (EU), is a U.S.–led initiative that strengthens critical mineral supply chains through cooperation with selected partners, representing the first institutionalized practice of the U.S.’s friend-shoring strategy.^8^ These developments might exacerbate the instability of the global rare earth supply chain, which calls for further study.

Existing research focuses on empirically analyzing the impact of geopolitical actions on rare earth price,^9^^,^^10^ supply chain resilience,^11^^,^^12^ gross trade value or volume,^13^ the supply–demand landscape,^14^^,^^15^ and trade network^16^ in the international market, elucidating the causal relationships between geopolitical factors and the resultant shifts in trade policy, thereby explicating their influence on the global rare earth market. Traditional empirical models and historical data analyses, while valuable for understanding past dynamics, are limited in their ability to assess the potential impacts of not-yet-enacted policy changes. In contrast, simulation-based approaches offer a promising avenue to address this gap, particularly for critical minerals such as rare earths, which are characterized by high uncertainty and policy sensitivity. Still, the existing simulation research on rare earths and other critical minerals remains relatively scarce. Dynamic Material Flow Analysis (DMFA) has been the main method for forecasting the supply and demand of rare earths in the future, yet it has rarely been applied to assess the impacts of policy disruptions on international markets.^17^ Some studies have sought to address this by employing system dynamics,^18^ game theory,^19^^,^^20^ and agent-based models^21^ to capture the interactive behaviors of market participants within shifting policy environments. For example, Nguyen and Imholte applied a system dynamics model to evaluate how changes in China’s rare earth policies affect both domestic and international demand.^18^ Brown and Eggert utilized game theory to examine how shifts in China’s policies influence illegal rare earth production and the supply responses of Australian mines.^19^ Riddle et al. employed the Argonne National Laboratory’s Global Critical Materials (GCMat) agent-based model to simulate how different regional rare earth supply disruptions could reshape trade flows between Chinese and non-Chinese markets.^21^

**Figure 1:**
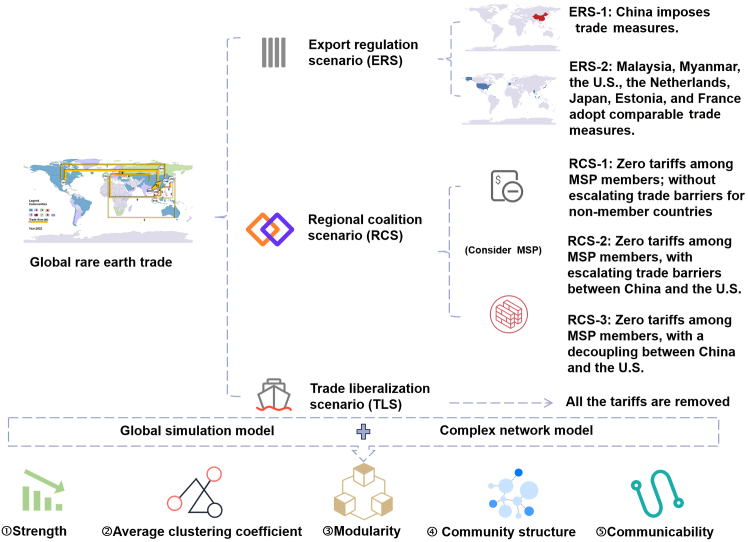
Research framework Figure 1 illustrates the analytical framework of this study. It encompasses a scenario design based on three distinct categories of geopolitical actions: implementing export regulations, forming regional coalitions, and pursuing trade liberalization; the latter serves as a more optimistic case. ERS and RCS are further divided into sub-scenarios, with full details provided in the STAR Methods section. Within the regional coalition scenarios, the Mineral Security Partnership (MSP) is used as the representative regional coalition for modeling purposes.

While these advances have significantly contributed to the field, important research gaps remain to be filled: (1) Most existing studies focus on the interactions of a limited number of economies, often treating non-Chinese markets as a single aggregated entity. While network-based models can reveal the impact of policy changes on individual countries, they generally omit price mechanisms.^22^ (2) Current simulation-based research on rare earths primarily centers around the implications of China’s policy shifts regarding global prices, supply security, and trade patterns, with insufficient attention being paid to potential disruptions originating from other economies. To bridge these limitations, this study aims to explore the potential effects of growing national geopolitical actions—in particular, the possible enforcement of trade barriers and the formation of regional coalitions by different economic entities—on the global rare earth trade network. A global simulation model incorporating price transmission mechanisms is combined with complex network analysis to investigate rare earth trade networks among 77 economies within three distinct scenarios: export regulation (ERS), regional coalitions (RCS), and trade liberalization (TLS) scenarios. The structural properties of the networks are analyzed using key metrics such as strength, average clustering coefficient, modularity, community structure, and communicability. Detailed scenario design and analysis methods are presented in the STAR Methods Section. By benchmarking these scenarios against the 2022 trade network (baseline scenario), this study provides a comprehensive evaluation of how trade barriers and regional coalitions—driven by potential geopolitical actions—reshape the global rare earth trade (Figure 1). Understanding such impacts is vital for policymakers to devise strategies that ensure the efficiency and stability of the rare earth supply chain, particularly during the critical period of global energy transition.

## Results

### Novel features of the global rare earth trade landscape

#### Newly prominent traders reduce export and import concentrations

**Figure 2:**
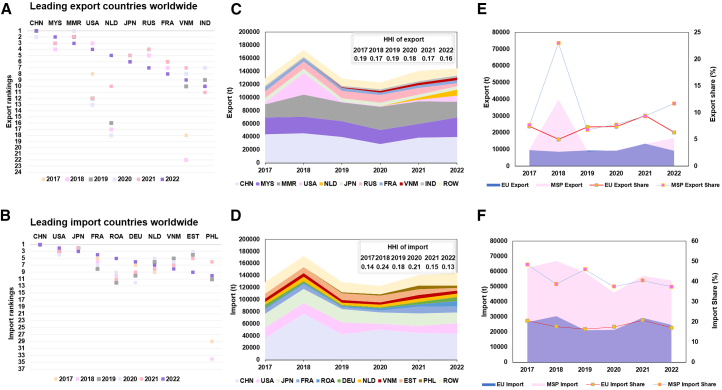
Import and export of major rare earth trading countries (2017–2022) (A) Rankings of top exporting countries for rare earths. (B) Rankings of top importing countries for rare earths. (C) Export volumes of the top countries. (D) Import volumes of the top countries. (E) Export volumes and their respective shares in global rare earths exports for MSP and EU. (F) Import volumes and their respective shares in global rare earths imports for MSP and EU. In Figure 2, HHI represents the Herfindahl–Hirschman Index, used to calculate the concentration of imports and exports. ROW refers to the rest of the world, excluding the countries in the corresponding figure. We display data for the remaining countries to highlight the share of major countries in the total export/import volume. Thus, the countries represented by the ROW in (C) and (D) are not the same. The U.S.’s rare earths exports in 2018 were significantly higher than those in other years, primarily due to an unusual surge in shipments to China. The names of countries and their corresponding ISO three-digit codes can be found in the Table S1.

In-strength and out-strength network nod attributes reflect a country’s export and import activities. Analysis of these metrics reveals that new leading trading entities have emerged on the international rare earth trade market, reducing both export and import concentrations (Figure 2). The concentration of imports has even entered a low-concentration state (<0.15).^23^ On the export side, although China, Malaysia, and Myanmar remained the top exporters of rare earths in 2022, it is noteworthy that the U.S., the Netherlands, and Vietnam, which had seldom ranked among the top exporters, rose to fourth, fifth, and ninth positions, respectively, in rare earths exports globally (Figure 2A). Meanwhile, Malaysia’s total exports also reached the highest level in the past six years (29,811 tonnes) (Figure 2C). This is the result of various countries' efforts to expand the refining and separation capacity of rare earths, including initiatives led by companies such as the U.S.'s MP Materials^24^ and Malaysia’s Lynas.^25^ The MSP coalition’s export volume and share increased in 2022, largely attributable to the growing contribution of non-EU members, particularly the U.S. (Figure 2E). On the import side, the Philippines and the Rest of Asian countries (ROA) have emerged as new top importers (Figure 2B), alongside France and Germany, increasing their share of total imports. Correspondingly, the combined import share of the three major countries—China, Japan, and the U.S.—declined from 60% in 2017 to 54% in 2022 (Figure 2D). Although the MSP saw its imports from non-member countries rebound after the COVID-19 pandemic, both the volume and share remained below pre-pandemic levels, likely reflecting the effects of friend-shoring. The EU, accounting for over 40% of MSP imports, experienced a comparable fluctuation (Figure 2F).

#### Transition of the rare earth trade network from globalization to regionalization

**Figure 3:**
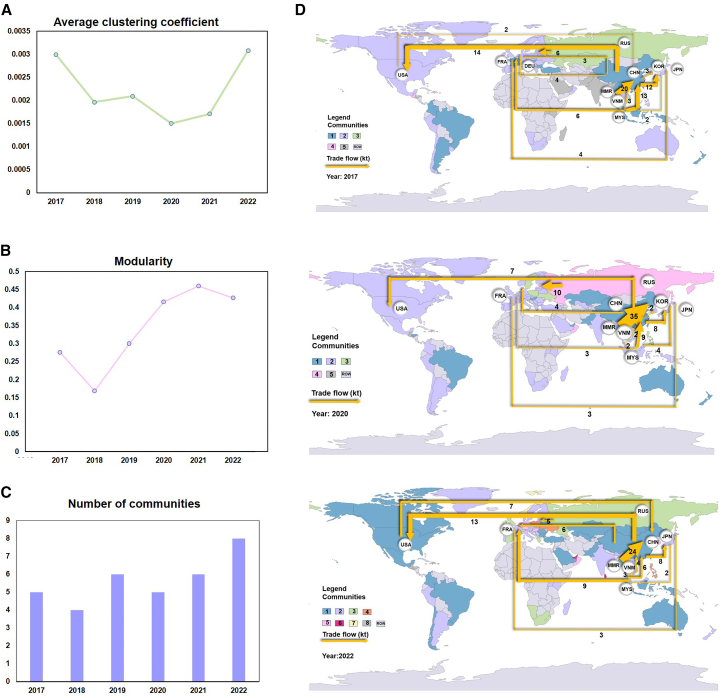
Structural characteristics of the rare earth trade network (2017–2022) (A) Average clustering coefficient of the rare earth trade network. (B) Modularity. (C) Number of communities. (D) Community structure and top rare earth trade flows. Figure 3D presents the community structure and top rare earth trade flows in 2017, 2020, and 2022. Countries with the same color belong to the same trade community, as identified through community detection analysis. This figure highlights the top 12 bilateral trade flows for each year, which together account for over 70% of the total trade volume. The labels “Community 1, 2,” and so forth, in the legend do not represent any specific ranking or order, but rather correspond to the sequence in which the communities were identified by the detection algorithm. Rare earth trade data from 2017 to 2022 are shown in Tables S2.

The overall network characteristics reflect the international trade landscape. The results indicate a shift in the rare earth trade network from globalization to regionalization. Between 2017 and 2022, the average clustering coefficient initially declined and then rebounded (Figure 3A). In 2017, strong trade links among major economies formed high-weight triangular relationships, leading to a high average clustering coefficient. However, the rise of trade protectionism and the COVID-19 pandemic weakened trade connections globally, resulting in their decline. As supply chains reorganized, many countries prioritized trade diversification while partnering with reliable regional allies—a trend known as friend-shoring.^8^ Although this shift weakened inter-regional linkages (e.g., China to Japan), it strengthened intra-regional connectivity (e.g., Malaysia to France) (Figure 3D), fueling the recovery of the average clustering coefficient. Rising modularity and an increasing number of communities further confirmed the growing prominence of regionalization (Figures 3B and 3C). Compared with 2017, the network in 2022 displayed significantly enhanced regionalization features. Figure 3D shows the community structure of the rare earth trade network for 2017, 2020, and 2022. It can be seen that Malaysia and Japan formed a regional community in 2022 as major trading countries. In the past, these countries were not independent and often formed a larger community with countries such as China or the U.S., which is further evidence of the regionalization of the rare earth trade market.

#### An emerging Asian trade community mediating China–U.S. relations

**Figure 4:**
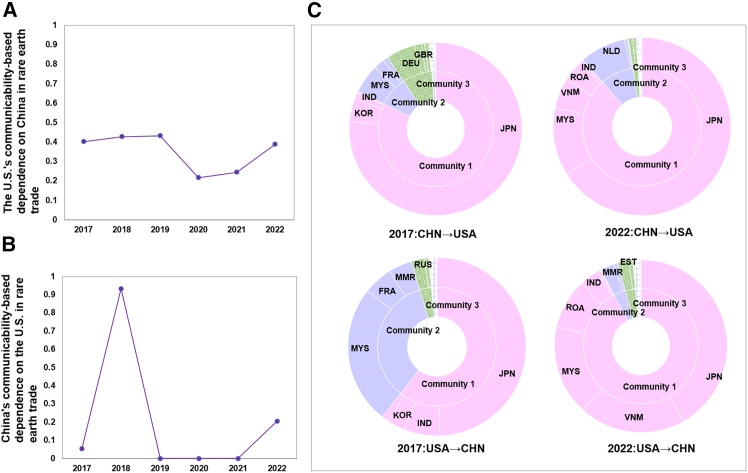
China–U.S. trade relations (A) The U.S.'s communicability-based dependence on China in rare earth trade (CHN→USA) (2017–2022). (B) China’s communicability-based dependence on the U.S. in rare earth trade (USA→CHN) (2017–2022). (C) The contribution of intermediary countries as trade bridges to the mutual dependence between China and the U.S in 2017 and 2022. Countries with the same color belong to the same community, as identified through community detection analysis.

The bilateral relationship between China and the U.S. within the rare earth trade structure is particularly noteworthy. Regarding the regionalization trend, Japan, Malaysia, Vietnam, and other Asian countries have formed a trade community with closer internal ties. Communicability analysis reveals that this group of countries is likely to play a growing balancing and mediating role in the evolving China–U.S. trade landscape. From 2017 to 2022, the U.S.'s communicability-based dependence on China for rare earth trade first declined and then rebounded (Figure 4A), while China’s communicability-based dependence on the U.S. peaked in 2018 and 2022 (Figure 4B). Although the mutual influence between China and the U.S. is primarily driven by their direct bilateral ties, the role of intermediary countries as trade bridges has been strengthened in recent years. Japan continues to be the most critical bridge in China–U.S. trade, but its contribution has declined, while Vietnam, Malaysia, and other Asian countries have steadily increased the role that they play. By 2022, Japan, Vietnam, Malaysia, and other Asian countries had formed a cohesive trade community. Compared with 2017, the rising share of their bridging roles may suggest an upward trend in the collective influence on China–U.S. trade dynamics (Figure 4C).

### International rare earth trade landscape within multiple scenarios

#### Impacts of export regulations by emerging suppliers

**Figure 5:**
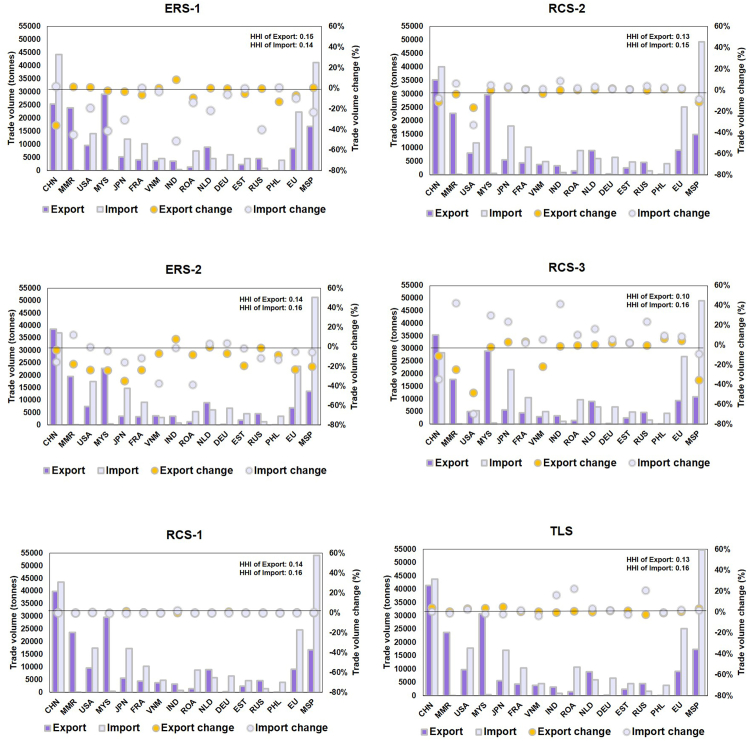
Rare earth trade of primary nations and coalitions in various scenarios The import/export variation rate refers to the degree of change relative to the BAU scenario (2022 baseline). These 14 nations collectively contribute to about 90% of the global import and export activities, thereby serving as a robust reflection of the core information.

Export taxes are employed to approximate the effects of export measures. Within ERS-1, China’s 25% export tax will reduce the global trade volume by 10.6% relative to the 2022 baseline. Within ERS-2, the simultaneous imposition of a 25% export tax by major Western and Asian exporters reduces the total trade volume by 12.4%. Sensitivity analyses with varying export tax rates confirm that regulations by emerging exporters can have significant impacts on global rare earth supply, highlighting the rising influence of diversified sources (Figure S1). While the aggregate global impact appears similar, the distributional effects differ markedly. In ERS-1, China’s export tax exerts a significant influence across a wide range of major rare earth-consuming regions. Imports decrease by 24% for the MSP as a whole, including a 19% decline in the U.S. and a 31% drop in Japan, which are the second- and third-largest importers, respectively. Within the EU, which sees an overall reduction of 10%, the Netherlands—being both an EU and MSP member—experiences a particularly sharp decline of 22%. Notably, China’s export reduction could exert less impact on global rare earth trade than in 2010, despite ERS-1 triggering a comparable 40% drop in Chinese exports. In 2010, a 40% cut in export quotas led to a 30% decline in global trade, with Japanese and U.S. imports falling by 44% and 43%, respectively. This reflects enhanced import diversification efforts—especially by Japan and the United States since 2010—to mitigate dependence on single nation. In ERS-2, the imposition of export taxes by Western and Asian exporters would have a narrow range of impact among importers. Chinese rare earths imports fall by 15%, from 43,569 to 37,082 tonnes, accounting for 35% of the total decline in global imports. As major demand centers, the MSP and EU experience only modest import reductions under ERS-2—just 5% overall—with U.S. imports remaining largely unaffected. France, however, is more significantly impacted, with its imports falling by 11%. Japan continues to face a notable decline of 16%, although the impact is less severe than under ERS-1 (Figure 5). The sharp decline in global rare earth imports in ERS-2 is primarily due to Myanmar’s export tax, which restricts access to heavy rare earths.^26^

#### Limited response of trade linkages to tariff elimination

Within RCS-1, the zero-tariff policy among MSPs increases the trade volume among coalition members by only 0.77%. This limited effect is primarily attributed to the fact that a substantial portion of the MSP coalition consists of EU member states, which already operate under a zero-tariff regime. Furthermore, this policy falls short of achieving the U.S.’s objective of decoupling from China. Although U.S. imports from MSP member countries increased by 2.7%, the reduction in imports from China is minimal. Overall, the global rare earth trade volume increased by just 0.1% relative to the 2022 baseline. In the TLS, the elimination of all current trade tariffs on rare earths will increase the global trade volume marginally by 2% compared with the 2022 baseline. As major exporters, China and Malaysia each expand their exports by 4%. Regarding imports, China’s rare earth imports rise by 1%, and the U.S.’s imports increase by 2%. The total demand from both the MSP and EU coalitions, considered collective entities, increases by 2%, respectively. Despite the limited response in trade volumes, the export concentration declines significantly under tariff elimination (TLS)—from 0.16 to 0.13—as more countries actively engage in global trade. In contrast, the decline under export regulation scenarios (ERSs) is relatively modest (0.14–0.15), reflecting a passive form of diversification driven by importers' efforts to seek alternative suppliers. A sharper reduction (to 0.10) occurs only under the U.S.–China decoupling scenario (RCS-3), where severe trade disruption forces a fundamental restructuring of supply relationships. However, this extreme case reflects reactive decoupling rather than sustainable diversification. Conversely, the import concentration rises in both cases: liberalization amplifies the share of major importers, while trade barriers reduce the demand from smaller economies, further concentrating imports among dominant players (Figure 5).

#### Trade barriers driven by geopolitical factors further strengthen trade regionalization

**Figure 6:**
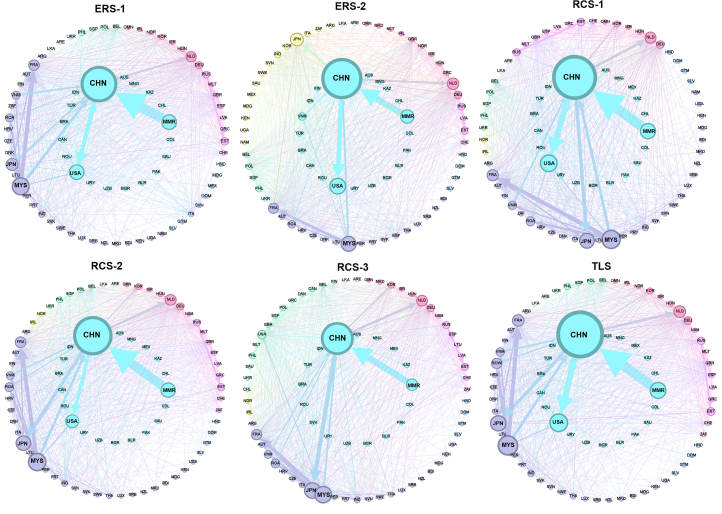
Community structure of the global rare earth trade network within different scenarios The community with the largest trade volume is shown in the inner circle. The node size reflects the cumulative trade volume of a given country or region; a larger node indicates a higher annual trade volume for that entity. Nodes of the same color are indicative of membership within a shared community. Edge width corresponds to the magnitude of trade flow, with wider edges denoting greater trade flow volume. The color of each flow aligns with that of its source. Table S3 outlines the trade network data for each scenario of the community analysis. Domestic production for domestic consumption is excluded from the community structure analysis.

The characteristics of the global trade network for rare earths within different scenarios indicate that the presence of trade barriers reinforces the regionalization of trade, leading to tighter regional cooperation. Compared with the baseline scenario, both the ERSs (where China or other major rare earth exporters introduce export measures) and RCSs (where trade barriers between China and the U.S. escalate) exhibit higher network modularity and average clustering coefficients (Table 1). This indicates that trade barriers hinder cross-regional trade while fostering intra-regional cooperation, resulting in a more defined community structure within the network. Tariff barriers reduce cross-regional triangular relationships but amplify intra-regional ones, with the latter outweighing the former, leading to an overall rise in the average clustering coefficient, except in ERS-1. Moreover, the global rare earth trade network exhibits two major trade communities: one centered around China, the U.S., and Myanmar, and the other around Japan and Malaysia. This two-community structure remains relatively stable within most scenarios. However, within scenarios where major non-Chinese suppliers impose export taxes, Japan and Malaysia diverge and form separate communities with different trade partners (Figure 6). Within RCS-3, heightened trade barriers between China and the U.S. similarly cause the two countries to split into distinct groups (Figure 6). These shifts also demonstrate that trade barriers reinforce the regionalization of trade networks. Core nodes that once coexisted within the same trade community tend to fragment under policy pressure, leading to a more decentralized trade landscape. In contrast, a liberalized trade environment promotes the deepening of globalization. Relative to the baseline scenario, the TLS (zero-tariff) and RCS-1 (zero-tariff within a trade coalition) exhibit a decrease in network modularity alongside an increase in the average clustering coefficient (Table 1). This indicates that the overall community structure of the network becomes less distinct. Cross-regional trade connections are strengthened, leading to greater interconnectedness across the network.

**Table 1:** Characteristics of the rare earth trade network within different scenarios

Scenario	Modularity	Average clustering coefficient	The U.S.' dependence on CHN	The CHN' dependence on U.S.
Baseline	0.427	0.003085	0.386971	0.206926
ERS-1	0.43	0.002648	0.282846	0.209437
ERS-2	0.429	0.003108	0.402242	0.170517
RCS-1	0.409	0.0031	0.386897	0.20673
RCS-2	0.431	0.003181	0.215492	0.149553
RCS-3	0.478	0.003852	0.023074	0.030306
TLS	0.426	0.003139	0.398262	0.214328

#### Only highly restrictive trade measures can disrupt trade interdependence

Focusing on the trade relationship between China and the U.S., only a significant increase in tariffs between the two countries would compel the U.S. to alter its current reliance on China. Within such a scenario, China and the U.S. would be separated into distinct trade groups, with their mutual dependence reduced to nearly zero (Table 1). Intensified trade barriers between China and the U.S. would result in a significant supply shortfall for the U.S., as MSP member countries are unable to compensate for the reduced imports from China. Within RCS-2, raising U.S.-China import and export tariffs to 25% would reduce U.S. imports from China from 13,426 tonnes to 7,478 tonnes. China would still remain the largest source of rare earths for the U.S., while imports from MSP member countries would increase by only 4%. Overall, the U.S. total imports would drop from 17,536 tonnes in the baseline scenario to 11,745 tonnes. Within RCS-3, a 200% tariff hike would drop the U.S. imports from China to 658 tonnes. Although imports from MSP member countries would see a marked increase of 14%, the total U.S. imports would decline sharply to 5,333 tonnes. The trade barrier threshold at which the U.S. and China no longer belong to the same trade community is reached when both countries impose approximately 180% reciprocal import and export tariffs on rare earths. Moreover, different tariffs between China and the U.S. would lead to a nearly identical reduction in the magnitude of China’s total exports. Within RCS-3, China’s exports to the U.S. are nearly 6,000 tonnes lower than those in RCS-2. However, increased exports to other countries, particularly Japan (rising from 8,257 tonnes in RCS-2 to 11,254 tonnes in RCS-3), keep China’s total exports at 88% of the baseline in both cases. This is likely due to the U.S. reducing their direct imports from China while other countries increase their imports from China to produce downstream products for the U.S. market. Evidently, U.S.–China decoupling is not easily achievable.

## Discussion

Studies conducted in the past often underestimated the potential impact of emerging rare earth suppliers, such as Myanmar, Malaysia, and the U.S., on the global market. Here, our study, based on GSIM and complex network apporach, have explored the impacts of those suppliers on global rare earth market. A particularly significant role is observed for Myanmar., which is currently a key supplier of heavy rare earths. Myanmar’s export faces significant uncertainties, including political instability and severe environmental damage during rare earth mining and separation.^26^ These factors pose substantial risks to the stability of other nations' rare earths imports.^27^ A shortage in the supply of heavy rare earths would likely trigger significant global price volatility, leading to increased uncertainty in production planning for permanent magnet manufacturers. Moreover, the possible trade barriers imposed by Malaysia and certain emerging suppliers should not be overlooked.

Trade barriers not only pose challenges for China but also risk fostering excessive regionalization, which could lead to a variety of negative consequences. First, discriminatory trade coalitions that reduce intra-regional trade barriers while escalating barriers with other regions are likely to cause over-regionalization. Within RCS-3, the decoupling of the U.S. and China forces the U.S. to rely on higher-cost suppliers or invest in developing its domestic rare earth processing industry.^28^ These substantial costs would ultimately be passed on to downstream products (such as electric vehicles, wind turbines, and electronic devices), increasing the financial burden on consumers in the long term. Simultaneously, the primary purpose of U.S. exports of rare earth ores to China is to leverage China’s processing capacity,^29^ and China’s rare earth supply chain products serve far more than just domestic demand. A significant reduction in the U.S.’s rare earths exports to China would therefore shift China to develop emerging supply chains form other nations, further diminishing the overall efficiency of the supply chain. Globally, excessive regionalization hinders optimal resource allocation, significantly reducing the efficiency of the rare earth supply chain. Secondly, excessive regionalization severely restricts technological collaboration between countries, slowing down innovation. Taking U.S.–China decoupling as an example, while China has extensive experience in the use of rare earth processing technologies, it may lose access to advanced manufacturing technologies such as chips and related technologies from the U.S.^30^; meanwhile, the U.S., lacking China’s expertise in rare earth smelting and separation,^31^ would face greater challenges and higher costs when attempting to achieve technological breakthroughs. If such trade barriers were extended to MSP members, the negative impacts would be even greater on global rare rath market. Finally, rising production costs of rare earths and a slowdown in technological innovation would weaken the competitiveness of renewable energy projects,^32^^,^^33^ suppress investment in renewable energy and electric vehicle production, and ultimately introduce uncertainty and delays to the global energy transition.

Trade liberalization and a non-discriminatory regional coalition can contribute to an overall increase in trade levels and enhance the attainability of rare earth resources. However, to effectively promote the application of rare earths and ensure the stability of the supply chain, the removal of tariffs alone may not yield favorable outcomes, necessitating the implementation of enhanced cooperation. In terms of the content of cooperation, cooperation breakthroughs in the recycling of rare earths and substitute materials can reduce conflicts and turbulence in the global trade market, thus ensuring a clean energy transition.^16^ In addition, widening supply chains through investment cooperation in new productive capacities is a good option. It is recommended that investment partners include a balanced mix of Western and non-Western investors, including, but not limited to, China.^34^ In terms of modes of cooperation, open regionalism provides a preferable framework for cooperation regarding critical raw materials. Unlike exclusionary approaches such as friend-shoring, open regionalism emphasizes institutional openness to non-members, adhering to non-discrimination principles and balancing efficiency with global value-chain stability. Its regulatory framework is transparent and adaptive, open to participation by any economy committed to following common rules.^35^ Open regionalism is not a new concept; existing agreements such as the Regional Comprehensive Economic Partnership (RCEP) and the Comprehensive and Progressive Agreement for Trans-Pacific Partnership (CPTPP) embody the logic of “deepening within, openness beyond”.^36^

Currently, key rare earth suppliers do not participate in the same open regional framework. Yet, existing research suggests that international cooperations could substantially enhance institutional attractiveness and the efficiency of cross-border resource allocation, benefiting all economies involved.^36^ However, there are significant differences between those suppliers regarding national security priorities and autonomy over critical supply chains, particularly in strategically sensitive sectors such as rare earths and semiconductors.^37^^,^^38^^,^^39^ These differences continue to limit the prospects for cooperation. Despite these challenges, the similarities between the key suppliers in terms of regulatory approaches toward low-carbon application for climate change mitigation offer feasible policy pathways for structured cooperation within the open regionalism framework.^40^^,^^41^ Three specific measures could be pursued: Firstly, cooperation boundaries should be clearly defined by categorizing products according to end-use sensitivity, prioritizing liberalization in low-risk sectors such as renewable energy and digital infrastructure. Secondly, a unified certification scheme should be established for trusted trade entities—firms with transparent end-use declarations and verified compliance records—to grant them preferential trading privileges. Thirdly, a neutral third-party audit mechanism should be implemented to verify end-use accuracy and monitor trade flows, thereby preventing unauthorized diversion to the restricted purposes. Together, these measures—applied within an open regional framework—would help to balance national security concerns with global supply chain stability while also shielding civilian sectors from the spillover effects of great-power competition and easing pressure on third-party countries to align with either side.

In summary, the global trade of rare earths is increasingly exhibiting a trend toward regionalization. Trade barriers and discriminatory regional coalitions further reinforce this trend, probably reducing the efficiency of the rare earth supply chain and delaying progress in the energy transition. To address these challenges, open regionalism and enhanced cooperation offer a viable solution, ensuring that both supply chain security and efficiency can be achieved.

### Limitations of the study

There are several sources of uncertainty in this simulation-based analysis. One example is the use of fixed and dated elasticity parameters. While this is common in trade policy evaluation,^42^^,^^43^ predictions of rare earth trade flows with the GSIM can be affected by alternative elasticity settings. Accordingly, we conducted a sensitivity analysis of the substitution elasticities (Table S4; Figures S2 and S3). Another uncertainty lies in the model’s exclusion of long-term developments such as secondary supply and material substitution, which are expected to reduce geopolitical influences on trade patterns over time. Nonetheless, the GSIM remains suitable for short-to medium-term trade simulations, as demonstrated by our historical validation comparing its simulation of China’s 2010 rare earth export quotas with actual trade outcomes (Figure S4).

The global simulation model (GSIM) employed in this study is suitable for performing policy simulations involving a single product, though it has limitations in terms of analyzing how policy adjustments driven by geopolitical actions propagate across different stages of the rare earth industry chain, including upstream (ores), midstream (oxides), and downstream (magnets). These challenges are also faced when using other modeling approaches to perform multi-level and multi-product quantitative analysis. In the future, we aim to develop an extended GSIM that incorporates multi-product linkage mechanisms to simulate how policy changes are transmitted along the rare earth supply chain. The integration of the extended GSIM with network analysis also opens up opportunities for further examination of community structures and the evolution of community centers within multilayer trade networks.^44^ This will enable a more comprehensive assessment of the stability of the global rare earth supply chain and the impact of related policies to take place.

## Resource availability

### Lead contact

Further information and requests for resources and materials should be directed to and will be fulfilled by the lead contact, Peng Wang (pwang@iue.ac.cn).

### Materials availability

This study did not generate any new physical materials.

### Data and code availability

• Data: this article analyzes existing, publicly available data that are listed in the key resources table.
• Code: this article does not report original code.
• All other items: any additional information required to reanalyze the data reported in this article is available from the lead contact upon request.

## Acknowledgments

This study was sponsored by the CAS IUE Research Program (No. IUE-JBGS-202202), the National Natural Science Foundation of China (No. 72274187, No. 42101286, No. 72304190, No. 42471337), the Zhejiang Provincial Natural Science Foundation of China (No. LQ22G030020 and Y21G030084), and the Zhejiang Office of Philosophy and Social Sciences Planning Project (No. 24NDQN175YBM).

## Author contributions

Y.W.H.: writing the original draft, reviewing and editing the draft, and data collection; P.W.: conceptualization, reviewing, and supervision; W.Q.C., Z.J.L. and W.J.S.: reviewing and visualization; and L.B.T., T.M.G., and H.C.H.: data collection and reviewing.

## Declaration of interests

The authors declare no competing interests.

## STAR★Methods

### Key resources table

**Table:** 

REAGENT or RESOURCES	SOURCE	IDENTIFIER
**Deposited data**		

Rare earth compounds (2846) trade data	CEPII-BACI database	https://www.cepii.fr/CEPII/en/bdd_modele/bdd_modele_item.asp?id=37
The tariffs and non-tariff measures of rare earth compounds	Market Access Map database of the International Trade Centre	www.macmap.org
Export restrictions on rare earth compounds	The inventory of export restrictions on Industrial Raw Materials provided by the Organization for Economic Co-operation and Development	https://www.oecd.org/en/publications/oecd-inventory-of-export-restrictions-on-industrial-raw-materials-2024_5e46bb20-en.html
Import substitution and export supply elasticities	Soderbery, A., 2018. Trade elasticities, heterogeneity, and optimal tariffs. J. Int. Econ. 114, 44–62.	https://doi.org/10.1016/j.jinteco.2018.04.008
Import demand elasticities	Reports by the Economic and Social Commission for Asia and the Pacific	https://www.unescap.org/resources/new-global-estimates-import-demand-elasticities-technical-note

**Software and algorithms**		

Gephi 0.10.	The Open Graph Viz Platform	https://gephi.org/
PyCharm	Jetbrains	https://www.jetbrains.com/pycharm/
The GSIM model		https://www.i4ide.org/content/wpapers.html

### Method details

To examine the impact of trade measures on the global rare earth trade landscape, we establish an integrated framework combining the Global Simulation Model (GSIM) with complex network analysis. GSIM generates simulated bilateral trade flows across different scenarios—including export regulation (ERS), regional coalition (RCS), and trade liberalization (TLS) scenarios—capturing how trade volumes between country pairs respond to policy shocks. These bilateral trade flow matrices are then converted into directed and weighted trade networks, where each node represents a country and each edge corresponds to the magnitude and direction of rare earth trade. This allows us to translate GSIM’s quantitative output into network form and apply complex network metrics (strength, average clustering coefficient, modularity, community structure, and communicability) to analyze the characteristics of the trade network. By comparing the simulated trade networks under each scenario with the 2022 baseline network, we can systematically assess the structural disruptions induced by geopolitical actions.

#### Multi-scenario simulation of the rare earth trade network

##### Global simulation model (GSIM)

GSIM is a multiregional computable partial equilibrium model developed and expanded by Francois and Hall.^45^ This study applies the GSIM to simulate world rare earth trade flow in the different trade scenarios. In this study, 2022 is set as the benchmark. A matrix of trade volume of benchmark, import tariff levels, export tax levels, and a series of supply, demand, and substitution elasticities for the rare earths of each country are input into the GSIM model to simulate a global rare earth market equilibrium. Specifically, different trade actions scenarios are simulated by adjusting export taxes or import tariffs for major exporting and importing countries. Rare earths are subject to different export taxes or import tariffs that affect their prices in both domestic and international markets. These prices, in turn, determine the trade flows of each country depending on how sensitive they are to price changes. The GSIM equates the changes in total import demand and total export supply to determine the international market equilibrium and then computes the new trade volumes of rare earths under different action scenarios. The resulting rare earth trade flows under these scenarios are compared with the baseline scenario to assess the impact of trade actions on trade flows. The GSIM can also estimates the effects of trade actions on rare earth prices, with results shown in Figure S5.

Compared to more complex Computable General Equilibrium (CGE) models, the GSIM requires less data and does not require detailed macroeconomic modeling. This allows it to perform effective policy analysis even when data resources are limited.^43^ The GSIM is relatively simpler than other trade models, with a highly structured framework that facilitates quick parameter adjustments and multi-scenario simulations. This simplicity makes the model more flexible in policy research and academic analysis, enabling it to adapt to rapidly changing policy environments.

The basic structure of the model comprises three key components: the development of relevant own-price and cross-price elasticities; the application of these elasticities to define changes in national supply and demand; and the specification of market clearing conditions.

###### Elasticities

We assume that, for each importer v, imports from exporter r ($M_{r}^{v}$) in a given product category (in this study, rare earths) depend on industry prices and total expenditure on that category:

(Equation 1) $$M_{r}^{v}=f\left(P_{r}^{v},P_{s,s\neq r}^{v},y^{v}\;\right)$$

where $y^{v}$ is total actual expenditure on imports of rare earths in country v, reflecting the total cost of domestic buyers. $P_{r}^{v}$ is the internal price for rare earths from region r within country v, and $P_{s,s\neq r}^{v}$ is the price of competing varieties from other regions. Internal price reflects the actual cost faced by domestic buyers when choosing among imports from different sources. It is based on the world price (Free on Board) and incorporates import-country measures such as tariffs, as well as export-country policies such as subsidies or export taxes.

Following Francois and Hall (1997), both cross-price and own-price elasticities ($N_{r,s}^{v},{\;N}_{r,r}^{v}$) are derived by differentiating Equation 1, applying the Slutsky decomposition to partial demand, and exploiting the zero-homogeneity property of Hicksian demand. The resulting expressions are as follows:

(Equation 2) $$N_{r,s}^{v}=\theta_{s}^{v}\left(E_{M}^{v}+E_{S}^{v}\right)$$

(Equation 3) $$N_{r,r}^{v}=\theta_{r}^{v}E_{M}^{v}-\sum_{s\neq r}\theta_{s}^{v}E_{S}^{v}=\theta_{r}^{v}E_{M}^{v}-(1-\theta_{r}^{v}{)E}_{S}^{v}$$

where $\theta_{s}^{v}$ is demand expenditure share (at internal prices). $E_{M}^{v}$ is the demand elasticity in importing region v. $E_{S}^{v}$ represents the substitution elasticity faced by the importing country *v* when sourcing rare earths from different exporting countries. It captures the extent to which importers can (and are willing to) shift between suppliers in response to relative price changes. $N_{r,s}^{v}$ denotes the price elasticity of import demand for rare earths in country *v*, measuring the responsiveness of imports from country *r* with respect to changes in the import price of country *s*. When *r*≠*s*, it captures the cross-price elasticity; when *r* = *s*, it reflects the own-price elasticity.

###### Changes in national supply and demand

Defining $P_{r}^{*}$ as the price at which exporter *r* offers rare earths on the world market, net of any export taxes or subsidies. $P_{r}^{v}$ as the internal price for the rare earths of country *v*, we can link the two prices as follows:

(Equation 4) $$P_{r}^{v}=P_{r}^{*}\left(1+t_{r}^{v}\right)\frac{1}{\left(1+s_{r}^{v}\right)}=P_{r}^{*}T_{r}^{v}\frac{1}{S_{r}^{v}}$$

In Equation 4, $T_{r}^{v}=1+t_{r}^{v}$ represents the tariff multiplier ($t_{r}^{v}$ is the ad valorem import tariff rate applied by country *v* on rare earths imported from country *r*.) $S_{r}^{v}=1+s_{r}^{v}$ serves as an export policy multiplier, reflecting the overall effect of export interventions on the effective export price. A positive value of $s_{r}^{v}$ corresponds to an export subsidy, whereby the exporter in country *r* receives a subsidy equal to a proportion $s_{r}^{v}$ of export revenue from sales to country *v*. Conversely, a negative value indicates an export tax, requiring the exporter to pay a tax equal to $\left|s_{r}^{v}\right|$ per unit of export revenue. When $s_{r}^{v}=0$, no export intervention policy is in effect.

Since exporters typically transact at free-on-board (FOB) prices, export supply to world markets is modeled as a function of the world price.

(Equation 5) $$X_{r}=f\left(P_{r}^{*}\right)$$

Equations 1, 4, and 5 can be differentiated and reformulated to produce the following expressions.

(Equation 6) $$\hat{M_{r}^{v}}=N_{r,r}^{v}\hat{P_{r}^{v}}+\sum_{s\neq r}N_{r,s}^{v}\hat{P_{s}^{v}}$$

(Equation 7) $$\hat{P_{r}^{v}}=\hat{P_{r}^{*}}+\hat{T_{r}^{v}}-\hat{S_{r}^{v}}$$

(Equation 8) $$\hat{X_{r}}=E_{X}^{r}\hat{P_{r}^{*}}$$

where $\hat{\;}$ denotes a proportional change, so that $\hat{x}=\frac{dx}{x}$. $E_{X}^{r}$ denotes the export supply elasticity of country *r*.

###### Market clearing conditions

Given the preceding set of equations, we further reformulate the model in terms of world prices. Specifically, by substituting Equations 7, 2, and 3 into Equation 6 and aggregating over all import markets, we derive Equation 9, which represents the proportional change in total imports from country *r* across all destinations.

$$\hat{M_{r}}=\sum_{v}\hat{M_{r}^{v}}=\sum_{v}N_{r,r}^{v}\hat{P_{r}^{v}}+\sum_{v}\sum_{s\neq r}N_{r,s}^{v}\hat{P_{s}^{v}}$$

(Equation 9) $$=\sum_{v}N_{r,r}^{v}\left[\hat{P_{r}^{*}}+\hat{T_{r}^{v}}-\hat{S_{r}^{v}}\right]+\sum_{v}\sum_{s\neq r}N_{r,s}^{v}\left[\hat{P_{s}^{*}}+\hat{T_{s}^{v}}-\hat{S_{s}^{v}}\right]$$

Based on the market clearing condition—that the proportional change in country *r*’s total exports must equal the proportional change in total imports from country *r* across all importing countries—we derive the following equation.

$$\hat{M_{r}}=\hat{X_{r}}\Rightarrow$$

(Equation 10) $$E_{X}^{r}\hat{P_{r}^{*}}=\sum_{v}N_{r,r}^{v}\left[\hat{P_{r}^{*}}+\hat{T_{r}^{v}}-\hat{S_{r}^{v}}\right]+\sum_{v}\sum_{s\neq r}N_{r,s}^{v}\left[\hat{P_{s}^{*}}+\hat{T_{s}^{v}}-\hat{S_{s}^{v}}\right]$$

Equation 10 serves as the core of the model. For any given set of *N* trading countries, the model yields exactly *N* market clearing conditions when domestic production for domestic consumption is included.

The parameters, calibrated coefficients, and endogenous variables used in the model are summarized in Table M1.

**Table:** Table M1 Notation

Indexes	
*r,s*	Exporting regions
*v*	Importing regions
**Parameters:** fixed, exogenous value	
$E_{S}^{v}$	The substitution elasticity $E_{S}^{v}$ indicates that in importing country *v*, a 1% change in the relative price between products from two exporting countries *r* and *s* will lead to an $E_{S}^{v}$% change in the relative import demand ratio, holding total expenditure constant.
$E_{M}^{v}$	$E_{M}^{v}$ denotes the aggregate import demand elasticity for country *v*. It reflects the percentage change in total imports when the composite price of the imported product changes by 1%, holding income constant.
$E_{X}^{r}$	$E_{X}^{r}$ represents the export supply elasticity of country *r*. It captures the percentage change in the quantity of exports supplied by country *r* in response to a 1% change in the export price, holding production conditions constant.
**Calibrated coefficients:** computed using parameters, identities, and base-year data	
$t_{r}^{v}$	$t_{r}^{v}$ is the ad valorem import tariff rate applied by country *v* on goods imported from country *r*
$T_{r}^{v}$	$T_{r}^{v}=1+t_{r}^{v}$, represents the tariff multiplier.
$s_{r}^{v}$	A positive value of $s_{r}^{v}$ corresponds to an export subsidy, whereby the exporter in country *r* receives a subsidy equal to a proportion $s_{r}^{v}$ of export revenue from sales to country *v*. Conversely, a negative value indicates an export tax, requiring the exporter to pay a tax equal to $\left|s_{r}^{v}\right|$ per unit of export revenue.
$S_{r}^{v}$	$S_{r}^{v}=1+s_{r}^{v}$, serves as an export policy multiplier.
$\theta_{r}^{v}$	$\theta_{r}^{v}$ denotes the share of country *v*’s import expenditure on a given product sourced from country r, evaluated at internal prices
$N_{r,s}^{v}$	Cross-price elasticity. The percentage change in imports of country *v* from *r* in response to a 1% change in the price of the product from competing exporter *s*.
$N_{r,r}^{v}$	Own price elasticity. The percentage change in imports of country *v* from country *r* due to a 1% increase in the price of imports from *r* itself.
**Endogenous variables**: determined within the model	
$\hat{P_{r}^{*}}$, $P_{r}^{*}$	$\hat{P_{r}^{*}}$ represents the proportional change in the export (FOB) price of goods from country *r* on the world market. The level of $P_{r}^{*}$ is world price for exports from region r. $P_{r}^{*}$ can be inferred from its proportional change $\hat{P_{r}^{*}}$ and the corresponding baseline data.
$\hat{P_{r}^{v}},P_{r}^{v}$	$\hat{P_{r}^{v}}$ represents the proportional change in internal prices of goods from region *r* imported into region *v.* This change is derived from the world price variation $\hat{P_{r}^{*}}$ and trade policy adjustments. The level of $P_{r}^{v}$ representing the internal price faced by importers in region v, can be inferred from $\hat{P_{r}^{v}}$ and the corresponding baseline data.
$\hat{M_{r}^{v}}$, $M_{r}^{v}$	$\hat{M_{r}^{v}}$ represents the proportional change in imports of a given product category by country *v* from country *r*, and is determined by the change in internal prices $\hat{P_{r}^{v}}$ .The level of $M_{r}^{v}$ denoting the import quantity, can be recovered from $\hat{M_{r}^{v}}$ and the baseline data.
$\hat{X_{r}}$, $X_{r}$	$\hat{X_{r}}$ represents the proportional change in exports from country *r* to world markets, determined by the change in the world price $\hat{P_{r}^{*}}$. The level of $X_{r}$ denoting the export supply from country *r*, can be recovered using $\hat{X_{r}}$ and the baseline data.

##### Scenario setting

To analyze the impact of trade measures, regional coalitions, and trade liberalization on rare earth trade networks, this study sets up three major scenarios: an export regulation scenario (ERS), a regional coalition scenario (RCS), and a trade liberalization scenario (TLS). The export regulation scenario includes two sub-scenarios, ERS-1 and ERS-2. This study adopts export taxes as a unified simulation instrument to capture the effects of various export measures—both tariff and non-tariff in nature. ERS-1 assumes that China indiscriminately levies an export tax on rare earths exported to other regions of the world. ERS-2 assumes that other major exporting countries simultaneously implement an equivalent export tax on rare earths exports. The regional coalition scenario is set against the backdrop of the MSP (Minerals Security Partnership),^8^ a prominent U.S.-led regional coalition aimed at enhancing the security of critical mineral supply chains. It includes three sub-scenarios. RCS-1 assumes that the main purpose of a regional coalition is to reduce trade barriers among its members. RCS-2 and RCS-3, considering the current U.S.–China rivalry, assume that the establishment of the MSP will initially lead to intensified trade barriers between the main member, the U.S., and the non-member, China. Specifically, RCS-2 reflects a scenario of conventional trade friction in the rare earth sector, while RCS-3 represents a more extreme decoupling scenario, in which China and the U.S. significantly reduce mutual trade in rare earth products. Finally, we set up a trade liberalization scenario (TLS), which removes all tariffs. Detailed information on each scenario is presented in Table M2.

**Table:** Table M2 Scenario settings

Scenario name	Detailed information
Business-as-usual (BAU)	We have designated the scenario in 2022 as the BAU scenario. In this scenario, the tariff values imposed by various countries on rare earths (2846) are at the 2022 level. At the same time, no country has adopted a rare earth export tax policy for rare earths in 2022.^46^
Export regulation scenario (ERS)	ERS scenario denotes the situation in which the principal exporting nation enforces export regulation measures. The ERS scenario consists of two sub-scenarios: ERS-1 and ERS-2. In ERS-1, China levies a 25% export tax on rare earths that are exported to other regions of the world. In ERS-2, Malaysia, Myanmar, the U.S., Japan, the Netherlands, Estonia and France apply a 25% export tax on rare earths that are exported to other regions of the world.
Regional coalition scenario (RCS)	For the RCS-1 scenario, we consider the Minerals Security Partnership (MSP) aiming to accelerate the development of diverse critical energy minerals supply chains for the U.S. The MSP is a collaboration of Australia, Canada, Estonia, Finland, France, Germany, India, Italy, Japan, Norway, the Republic of Korea, Sweden, the United Kingdom, the United States, and the European Union (represented by the European Commission).^47^ There will be no trade barriers for rare earth trade among the member countries.
	In RCS-2, China and the U.S. each impose a 25% tariff on rare earths imports from the other, and both countries also impose a 25% export tax on rare earths they export to each other. To secure its rare earth supply, the U.S. relies on the MSP coalition, where all members agree to eliminate tariffs and non-tariff barriers on rare earth trade among themselves. In RCS-3, the import and export taxes between China and the U.S. rise to 200%, and the U.S. still relies on the MSP to cope with the higher rare earth costs.
Trade liberalization scenario (TLS)	The TLS assumes that all the tariff and non-tariff measures are removed, which is designed to test whether an environment of trade liberalization can facilitate global rare earth trade.

The quantification of trade barrier impacts, the justification for scenario design, and the exclusion of recycling and secondary supply considerations are further explained as follows.

First, the quantification of trade barriers. Trade barriers are categorized into tariff and non-tariff measures (NTMs). The GSIM can directly capture tariff changes (import tariffs and export taxes). Non-tariff measures (NTMs) can generally be categorized into import-side and export-side measures.^48^ For HS 2846 (rare earth compounds), import NTMs recorded in the UNCTAD TRAINS database are mainly sanitary and phytosanitary (SPS) and technical barriers to trade (TBT) measures,^49^ whose ad valorem equivalents (AVEs) are typically below 2%^50^—equivalent to modest tariffs—and thus have limited impact on trade flows. Moreover, importing countries rarely impose high barriers on rare earths given their strategic importance. Given these factors, our analysis does not focus on changes in import NTMs. By contrast, export-side NTMs (e.g., export quotas, licenses, technology restrictions) are strategically motivated and flexibly adjusted, making them difficult to model systematically. For instance, China reduced rare earth export quotas from 50,000 to 30,000 tons in 2010 and imposed different export controls in 2025.^51^^,^^52^ Since these measures ultimately reduce export volumes in a manner similar to export taxes, we follow prior studies^53^ in representing export NTMs through adjustments in export tax rates, which capture their primary effect on the global rare earth trade network.

Second, the rationale for scenario design. To assess how export measures affect the global rare earth trade network, we design ERSs. In ERS-1, China is modeled to impose a 25% export tax on rare earths. This scenario is inspired by China’s important role and past use of export controls.^54^ Historically, China applied export taxes of 2–25%^55^ and cut export quotas by 40% in 2010,^51^ triggering major disruptions. Model estimates suggest that a 25% export tax would lead to a similar 40% export reduction of China, enabling assessment of how a unilateral barrier from the major supplier could reshape global trade. In ERS-2, we simulate a coordinated imposition of 25% export taxes by Malaysia, Myanmar, the United States, Japan, the Netherlands, Estonia, and France. These countries are selected because (1) Malaysia and Myanmar have announced or considered export restrictions^56^^,^^57^; (2) countries such as Japan, Malaysia, France, and Estonia possess refining and separation capabilities^58^; (3) Malaysia, Myanmar, Japan, the U.S., and the Netherlands ranked among the major exporters of rare earth compounds in 2022. In the RCSs, we use the MSP as a representative alliance. In RCS-1, zero tariffs are applied among MSP members. However, the security of critical mineral supply, including rare earths, provided by MSP member countries allows the U.S. to pursue heightened trade barriers with other countries, particularly with China. Both countries are also engaging in broader technological competition, including mutual restrictions on access to critical technologies.^59^^,^^60^ Based on these dynamics, in RCS-2, we simulate reciprocal 25% import tariffs and export taxes between the U.S. and China to reflect rising tensions. RCS-3 explores the threshold for decoupling: model tests show that at around 180% tariffs and export taxes—serving as a theoretical boundary rather than a projection of actual policy—the two countries no longer belong to the same trade community. To simulate a near-complete decoupled scenario, we set the rate at 200%.

Third, considerations regarding recycling and secondary supply. Scenario design does not consider the impact of rare earth recycling and secondary supply on the global trade network. Currently, most rare earth recycling feedstock originates from manufacturing losses,^61^ while recovery from end-of-life (EOL) consumer products remains minimal due to low collection rates, unfavorable economics, and the absence of cost-effective recovery technologies.^62^^,^^63^ As a result, countries without domestic production capacity face significant barriers to sourcing rare earths through recycling. In addition to secondary supply strategies, rare-earth substitution has also been investigated. Rare-earth-free magnets, including ferrite, manganese-based, and AlNiCo types, have been explored as potential substitutes.^64^ However, substitution remains challenging across the supply chain. While alternative materials are available, they generally fall short in performance and cost-effectiveness, especially for critical applications in clean energy technologies.^65^ Taken together, secondary supply and substitution are unlikely to alter global rare earth trade patterns in the short to medium term. However, should these strategies mature and scale, the criticality of rare earths may diminish over time—potentially fostering a more liberalized trade environment.^16^

##### Sensitivity analysis and model validation

The projection results of rare earth trade flows with GSIM model can also be affected by varied export tax levels and substitution elasticity settings, etc. To evaluate the robustness of global supply under varying regulatory conditions, we simulate four export tax levels (10%, 25%, 50%, and 100%) imposed either by China or by major non-Chinese exporters (Figure S1). In this context, “major non-Chinese exporters” refers to the set of countries identified in the ERS-2 scenario as major rare earth suppliers excluding China. For each scenario, we further assess the sensitivity of trade outcomes by adjusting the substitution elasticity (SE) by ±10% relative to the baseline, to examine the sensitivity of projected total trade volumes and country-level import and export responses (Table S4; Figures S2 and S3).

To further assess the accuracy of the Global Simulation Model (GSIM), we evaluated its performance in replicating trade responses to policy-induced shocks, using China’s rare earth export control introduced in 2010 as a test case. That year, China reduced its rare earth export quota from 50,000 tonnes in 2009 to 30,000 tonnes by 2011.^51^ We used the GSIM model to simulate the effects of this policy shock by adjusting export tax rates to reduce China’s simulated export volume to the actual level observed in 2012. We selected 2012 as the validation year for two main reasons. First, it is important to note that GSIM produces equilibrium outcomes and may not fully capture price shocks. Following China’s export measures, rare earth prices experienced extreme volatility—rising sharply and then falling before stabilizing around 2012.^66^ Second, other countries began implementing policy responses, such as the United States restarting domestic production, with the Mountain Pass mine resuming commercial operations in 2017.^67^ These measures, however, typically have delayed impacts. In short, we benchmarked the GSIM simulation results against actual trade data of 2012, a year when markets had largely stabilized and distortions from other countries’ policy responses were relatively limited. We compared the simulated and observed import and export volumes of key countries, and assessed the similarity between the simulated and actual global trade matrices using correlation coefficients. This allowed us to evaluate the reliability of the GSIM model in trade responses to policy changes (Figure S4).

#### Analysis of trade network features

Complex network analysis is used to analyze the structure of the global rare earth trade network, which is formalized as a weighted directed graph ***Gr=(V, E,W).*** Each node *v∈****V*** corresponds to a country, and each directed edge $e_{ij}$ *∈* ***E*** from country *i* to country *j* represents an export flow, with the edge weight $w_{ij}$ *∈* ***W*** indicating the trade volume between the two countries. The node characteristics of the network are measured by out-strength and in-strength, whereas the network’s overall structural features are analyzed by the average clustering coefficient, modularity, community structure and communicability.^68^^,^^69^^,^^70^^,^^71^

##### In-strength and out-strength

Strength reflects the total amount of trade in which a country engages. The more trades a country performs, the more influence it has on the global rare earth market when its supply or demand changes. In-strength, $s_{i}^{in}$, and out-strength, $s_{i}^{out}$, indicate how much a country imports and exports, respectively. These values can be computed using Equations 11 and 12.

(Equation 11) $$s_{i}^{in}=\sum_{j=1}^{N}w_{ji}$$

(Equation 12) $$s_{i}^{out}=\sum_{j=1}^{N}w_{ij}$$

where $w_{ji}$ is the volume of rare earths that country *i* imports from country *j*, $w_{ij}$ is the volume of rare earths that country *i* exports to country *j* and *N* is the total number of nodes/country in the network.

##### Average clustering coefficient

The clustering coefficient in a weighted directed network, in the context of international trade, measures the extent to which a country’s trade partners also trade with each other, as well as the intensity of those trade relationships (measured by trade volume or value). The clustering coefficient of country i (${CC}_{i}$) is defined by Equation 13.

(Equation 13) $${CC}_{i}=\frac{1}{k_{i}\left(k_{i}-1\right)}\sum_{j,k}{\left({\hat{w}}_{ij}{\hat{w}}_{ik}{\hat{w}}_{jk}\right)}^{1/3}$$

where $k_{i}$ is the out degree of country *i*. ${\hat{w}}_{ij},{\hat{w}}_{ik}$ is the trade flow from *i* to *j*, from *i* to *k* respectively, normalized to a value between [0,1], typically achieved by dividing by the maximum trade volume in the network. ${\hat{w}}_{jk}$ represent the trade volume from trade partner *j* to partner *k*. The exponent 1/3 is used to ensure consistency in units when multiplying the weights.

The average weighted clustering coefficient is the average of the clustering coefficients across all nodes/countries:

(Equation 14) $$\bar{CC}=\frac{1}{N}\sum_{i=1}^{N}{\;CC}_{i}$$

##### Modularity and community detection

The modularity is used to determine the network community structure. Countries within the same community engage in more intensive trade than with outside countries. The optimal community partition for each node/country can be obtained by maximizing the modularity, *Q*, which is calculated using Equation 15.

(Equation 15) $$Q=\frac{1}{W}\sum_{ij}\left(w_{ij}-\frac{s_{i}^{out}s_{i}^{in}}{W}\right)\delta \left(C_{i},C_{j}\right)$$

where W is the total trade volume of all links in the network, $C_{i}$ and $C_{j}$ are the communities in which country i and j are located, and $\delta \left(C_{i},C_{j}\right)$ is 1 if i and j are in the same community, and 0 if they are not.

The combined analysis of the average clustering coefficient and modularity provides valuable insights into the structural changes of a trade network. An increase in the average clustering coefficient typically indicates a rise in triangular relationships, reflecting tighter trade connections between nodes. Meanwhile, an increase in modularity signifies a more pronounced modular structure, with clearer community divisions. When both metrics increase within the rare earth trade network, it suggests a trend toward regionalization. Here, regionalization does not refer to geographic proximity, but to strategic alignment and trusted partnerships. In this situation, intra-regional trade connections become stronger, while inter-regional connections weaken. However, the increase in intra-regional connectivity outweighs the decrease in inter-regional connectivity, resulting in an overall rise in the average clustering coefficient. Conversely, if modularity decreases while the average clustering coefficient increases, it points to a trend toward globalization. A decline in modularity indicates less distinct community divisions, with stronger inter-regional connections and potentially weaker intra-regional connections. Nevertheless, the enhancement of inter-regional connectivity surpasses the reduction in intra-regional connectivity, leading to an overall increase in the average clustering coefficient.

##### Communicability

The concept of communicability between nodes describes the ability of information or influence to be transmitted from one node to another. It considers not only direct connections but also the ability to transmit information through multi-step paths, thereby reflecting the overall level of association between two nodes.^72^ In this study, communicability is used to measure the mutual dependence between countries in the trade network. The adjacency matrix ***W*** is defined, where represents the trade flow from country *i* to country *j*. The communicability matrix ***G*** is calculated using the matrix exponential of ***W***:

(Equation 16) $$G=\left(G_{ij}\right)=e^{W}=\sum_{k=0}^{\infty }\frac{W^{k}}{k!}$$

Here, $G_{ij}$ quantifies the overall strength of direct and indirect trade connections between country *i* and country *j*, with higher values indicating stronger trade ties. Given the critical role of U.S.-China relations in rare earth trade, if *i* represents China and *j* represents the U.S., $G_{ij}$ reflects the U.S.' dependence on rare earths from China, while $G_{ji}$ represents China’s dependence on the U.S.

In addition to the direct trade relationship between China and the U.S., third countries also play a supporting role in their mutual dependence by serving as intermediaries. The supporting effect of a given country *i* on the U.S. dependence on China can be measured by Equation 17.

(Equation 17) $$C_{CHN\to i\to USA}=G_{CHNi}\times G_{iUSA}$$

In the equation, $G_{CHNi}$ denotes the total communicability from China to the intermediary country *i*, and $G_{iUSA}$ represents the total communicability from country *i* to the U.S.

#### Data processing

Rare earth compounds, which are midstream products in the rare earth industry chain, are chosen as the focus of this study. Firstly, rare earth compounds account for a significant portion of trade volume among rare earth products (including rare earth ores, rare earth compounds, and rare earth metals).^73^ Secondly, co-opetition between major countries in the rare earth sector are primarily concentrated on rare earth compounds. The U.S., Japan, and European countries are particularly focused on developing midstream processing capabilities in the rare earth industry.^58^ To analyze the characteristics of the current rare earth trade network, we use data from 2017 to 2022 for the following reasons. First, 2018 marked the intensification of U.S.–China trade tensions, which significantly reshaped global supply chain strategies. Second, following the COVID-19 outbreak in late 2019, a marked rise in trade protectionism began to influence rare earth flows and policy coordination. Third, since 2017, the U.S., the EU, and Japan have accelerated the rollout of national critical mineral strategies, which include the diversification of rare earth supply sources and increased stockpiling and investment initiatives. These geopolitical pressures have collectively driven structural shifts in the global rare earth trade network.

Rare earth compounds (2846) trade data is available from the CEPII-BACI database,^73^ which is based on UN COMTRADE data but further cleaned and refined. The original BACI dataset on rare earth oxides includes trade data for 235 economies. We excluded countries with virtually no import or export volumes. The share of deleted data is minimal and does not affect the robustness of the results. Ultimately, we retained trade data for 77 economies. The Market Access Map database of the International Trade Centre^74^ reports on the tariffs and non-tariff measures of rare earth compounds. Export restrictions on rare earth compounds are from the inventory of export restrictions on Industrial Raw Materials provided by the Organization for Economic Co-operation and Development.^46^ Import substitution and export supply elasticities are taken from Soderbery.^75^ Finally, new global estimates of import demand elasticities are taken from reports by the Economic and Social Commission for Asia and the Pacific.^76^ For countries lacking elasticity data, we substituted missing values using those of countries with comparable economic scale and geographic location. The processed data for rare earth trade (2017–2022), tariff (2022), export tax (2022), elasticities (estimated in existing literature), and simulation results of the global rare earth trade network under different scenarios are provided in Tables S2, S3, S5, and S6.

### Quantification and statistical analysis

This study does not include statistical analysis or quantification.

## Supplemental information

Document S1. Figures S1–S5 and Tables S1, S4 and S6

Table S2. Rare earth trade data from 2017 to 2022

Table S3. Rare earth trade network data for each scenario

Table S5. Import tariff and export tax implemented by each country under the baseline scenario
