# Supplementary material for: Exploring the divergence of rare earth trade networks with a global simulation model
Source: iScience. 2025 Sep 27;28(11):113658. doi: 10.1016/j.isci.2025.113658 (PMC12554209; doi:10.1016/j.isci.2025.113658)
Supplement: Document S1. Figures S1–S5 and Tables S1, S4 and S6 [file mmc1.pdf]

iScience, Volume *28*

Supplemental information

Exploring the divergence of rare earth trade networks with a global simulation model

Yawen Han, Peng Wang, Zhongju Liao, Linbin Tang, Wenjuan Song, Tianming Gao, Hongchang Hao, and Wei-qiang Chen

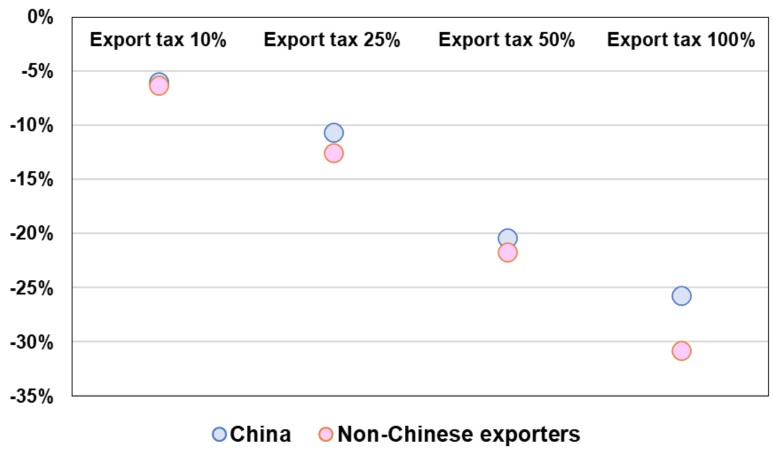

Figure S1. Projected changes in global rare earth supply relative to the 2022 baseline under different export tax rates (10%, 25%, 50%, and 100%) imposed by China and major non-Chinese exporters, related to STAR Methods

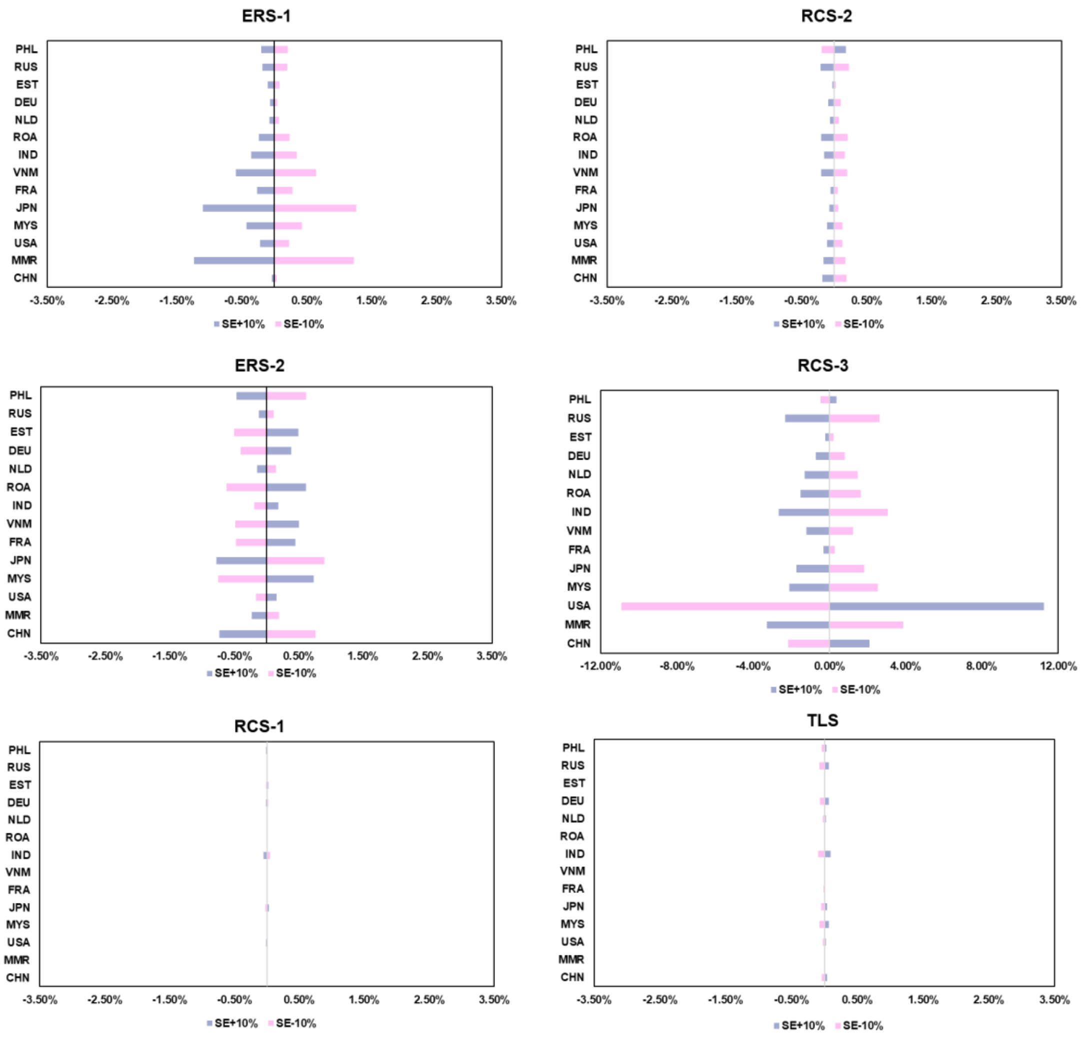

Figure. S2 Sensitivity of rare earth import in 14 countries/economies under different scenarios with substitute elasticity (SE) ranging from -10% to +10%, related to STAR Methods

Note: Percentages show changes compared with imports when SE is kept at its original value. A ±10% variation in substitution elasticity generally results in modest changes (within ±1.5%) in import volumes for major countries. However, under the RCS-3 scenario—characterized by high trade barriers between China and the U.S.—such elasticity shifts lead to larger impacts, with variations around 3% for major trading nations, and the U.S. experiencing changes exceeding 10%. This indicates that elevated trade barriers amplify the effect of substitution elasticity, particularly for the U.S., which increasingly turns to alternative suppliers under higher elasticity settings.

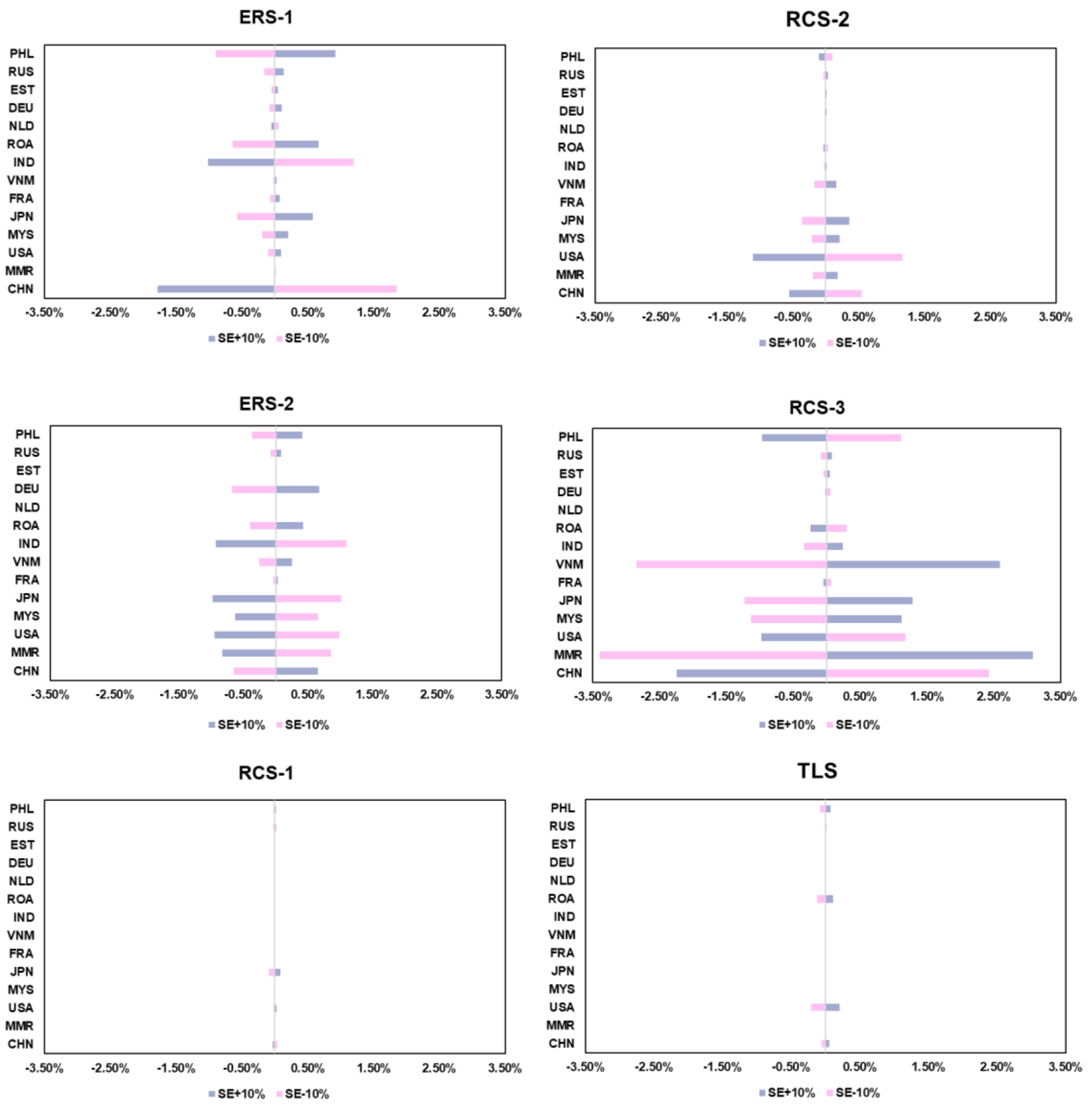

Figure. S3 Sensitivity of rare earth export in 14 countries/economies under different scenarios with substitute elasticity (SE) ranging from -10% to +10%, related to STAR Methods

Note: Percentages show changes compared with exports when SE is kept at its original value. The figure indicates that a ±10% variation in import substitution elasticity has a relatively minor impact on the export volumes of major trading nations, with fluctuations generally within 3.5%.

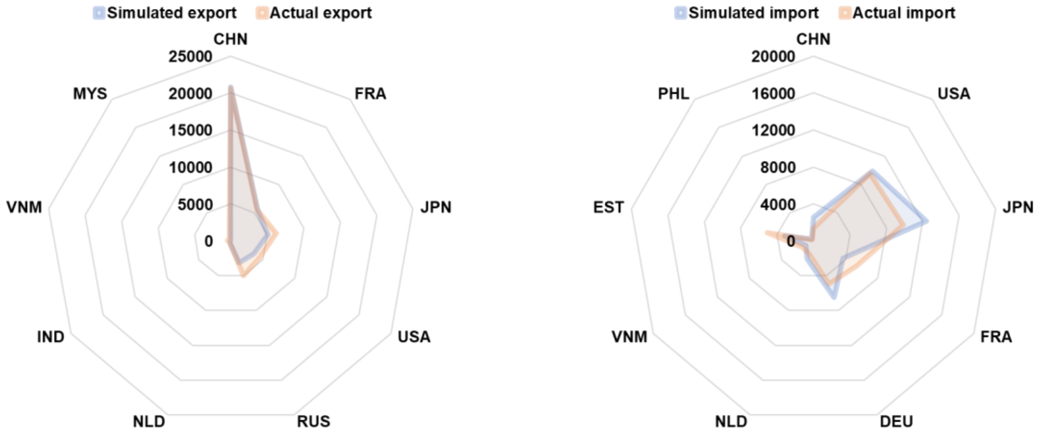

Figure S4. Comparison of simulated and actual import and export volumes for major countries (tonnes), related to STAR Methods

Note: The simulated import and export volumes of major countries are reasonably approximate the actual data. The similarity between the simulated trade matrix and the 2012 actual trade matrix was calculated to be 0.7636, underscoring the reliability of the GSIM model in capturing the global trade impact of China's export measures.

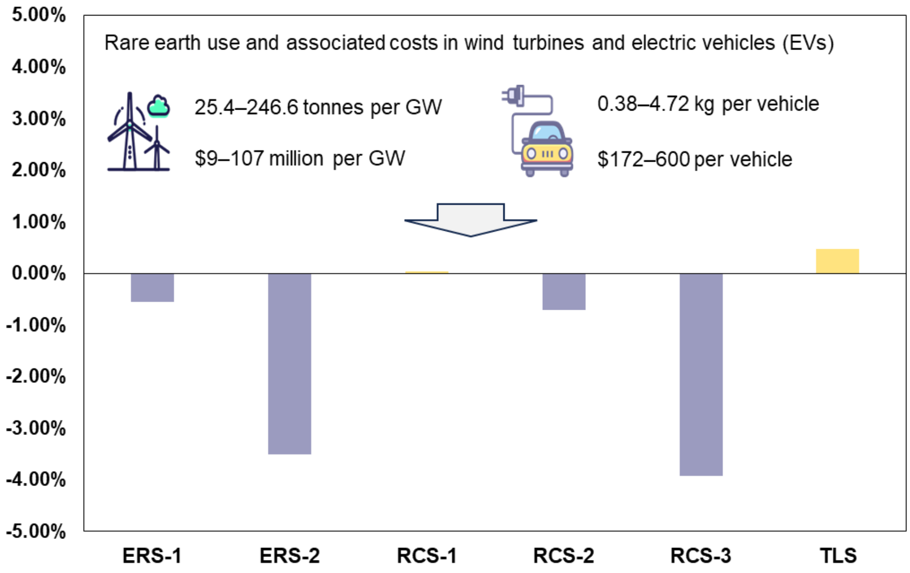

Figure S5. Rare-earth use and costs in wind turbines and electric vehicles, along with rare-earth price/cost variations across different scenarios relative to the 2022 baseline, related to STAR Methods

Note 1: Cost variations are assumed equal to changes in international equilibrium prices, which remain modest (<5%) and reflect differences between pre- and post-policy equilibrium rather than short-term volatility.

Note 2: The model yields a counterintuitive insight: trade barriers—regardless of their origin—can slightly reduce global prices, whereas liberalization leads to limited price increases. Crucially, price declines neither improve export revenues nor reduce input costs for downstream industries. Historical experience shows that demand often fails to rebound after price drops, limiting export gains[1]. For import-dependent firms, trade barriers increase policy uncertainty, causing delays in procurement and investment[2,3]. Rather than responding solely to price signals, firms pursue structural adjustments—such as diversification and substitution[4,5]—which are costly and time-consuming[4,6]. A slight price increase, by contrast, is too small to impose cost pressure but may reflect stable demand and policy conditions, supporting investor confidence and efficient resource allocation.

Note 3: The estimated rare earth cost per electric vehicle and per gigawatt of wind capacity is derived by converting the required quantities of individual rare earth metals into their corresponding oxide equivalents and applying market prices for the respective oxides[7,8].

Table S1. ISO three-digit country codes, related to Figure 2

country_name_full	iso_3digit_alpha	num region	country_name_full	iso_3digit_alpha	num region
Argentina	ARG	1	Malaysia	MYS	40
Australia	AUS	2	Malta	MLT	41
Austria	AUT	3	Mexico	MEX	42
Belgium	BEL	4	Oman	OMN	43
Brazil	BRA	5	Namibia	NAM	44
Bulgaria	BGR	6	Netherlands	NLD	45
Myanmar	MMR	7	New Zealand	NZL	46
Burundi	BDI	8	Norway	NOR	47
Belarus	BLR	9	Pakistan	PAK	48
Canada	CAN	10	Peru	PER	49
Sri Lanka	LKA	11	Philippines	PHL	50
Chile	CHL	12	Poland	POL	51
China	CHN	13	Portugal	PRT	52
Colombia	COL	14	Romania	ROU	53
Croatia	HRV	15	Russian Federation	RUS	54
Czechia	CZE	16	Saudi Arabia	SAU	55
Denmark	DNK	17	Serbia	SRB	56
Dominican Republic	DOM	18	India	IND	57
El Salvador	SLV	19	Singapore	SGP	58
Estonia	EST	20	Slovakia	SVK	59
Finland	FIN	21	Viet Nam	VNM	60
France	FRA	22	Slovenia	SVN	61
Germany	DEU	23	South Africa	ZAF	62
Greece	GRC	24	Spain	ESP	63
Guatemala	GTM	25	Sweden	SWE	64
Honduras	HND	26	Switzerland, Liechtenstein	CHE	65
Hungary	HUN	27	Thailand	THA	66
Indonesia	IDN	28	United Arab Emirates	ARE	67
Ireland	IRL	29	Turkey	TUR	68
Israel	ISR	30	Uganda	UGA	69
Italy	ITA	31	Ukraine	UKR	70
Japan	JPN	32	Macedonia	MKD	71
Kazakhstan	KAZ	33	United Kingdom	GBR	72
Kenya	KEN	34	the United States	USA	73
Republic of Korea	KOR	35	Uruguay	URY	74
Latvia	LVA	36	Uzbekistan	UZB	75
Lithuania	LTU	37	Mongolia	MNG	76
Luxembourg	LUX	38	Rest of Asia	ROA	77
Madagascar	MDG	39			

Table S4. Sensitivity of rare earth trade volume under different scenarios with substitute elasticity (SE) ranging from -10% to +10%, related to STAR Methods

	Trade volume (tonnes)			Trade volume change	
	SE	SE+10%	SE-10%	SE+10%	SE-10%
ERS-1	134247.2	133878	134644	-0.275%	0.296%
ERS-2	132956.8	132768.1	133177.9	-0.142%	0.166%
RCS-1	149418.1	149411.1	149424.6	-0.005%	0.004%
RCS-2	142053.4	141891.4	142217.5	-0.114%	0.115%
RCS-3	133293.6	133357.5	133297.5	0.048%	0.003%
TLS	152411.5	152452.7	152368.6	0.027%	-0.028%

Table S6. Import demand elasticity, export supply elasticity, and import substitution elasticity of each country, related to STAR Methods

Country	Import demand elasticity	Export supply elasticity	Import substitution elasticity	Country	Import demand elasticity	Export supply elasticity	Import substitution elasticity
ARG	-3.2324	5.2986	1.4290	MYS	-3.2324	5.2986	3.8575
AUS	-0.5411	5.2986	1.4290	MLT	-3.2324	5.2986	1.4290
AUT	-3.2324	5.2986	1.4290	MEX	-3.2324	5.2986	7.9684
BEL	-3.2324	0.3380	1.4290	OMN	-3.2324	5.2986	1.4290
BRA	-0.4666	5.2986	2.7466	NAM	-3.2324	5.2986	1.4290
BGR	-3.2324	5.2986	1.4290	NLD	-0.1915	0.7938	1.4290
MMR	-3.2324	5.2986	1.4290	NZL	-0.5000	5.2986	1.4290
BDI	-3.2324	5.2986	1.4290	NOR	-3.2324	5.2986	1.4290
BLR	-8.6544	5.2986	1.4290	PAK	-6.6735	5.2986	1.4290
CAN	-4.9056	0.1534	3.9639	PER	-3.2324	5.2986	1.4290
LKA	-3.2324	5.2986	1.4290	PHL	-1.2262	5.2986	2.5514
CHL	-3.2324	5.2986	1.4290	POL	-1.1144	0.0174	4.6710
CHN	-3.2324	4.7840	0.7887	PRT	-3.2324	5.2986	0.1029
COL	-3.2324	5.2986	1.4290	ROU	-3.2324	5.2986	1.4290
HRV	-3.2324	5.2986	1.4290	RUS	-3.2324	0.1762	1.2885
CZE	-0.3617	5.2986	1.0942	SAU	-3.2324	5.2986	0.0160
DNK	-3.2324	5.2986	1.4290	SRB	-3.2324	5.2986	1.4290
DOM	-3.2324	5.2986	1.4290	IND	-3.2324	0.0067	2.1023
SLV	-3.2324	5.2986	1.4290	SGP	-3.2324	0.0201	3.5600
EST	-3.2324	1.2004	1.4290	SVK	-3.2324	5.2986	1.4290
FIN	-3.2324	5.2986	1.4290	VNM	-3.2324	5.2986	1.4290
FRA	-0.0418	1.1691	1.5715	SVN	-3.2324	5.2986	1.4290
DEU	-1.3453	5.1403	3.4809	ZAF	-4.0196	0.0236	0.2024
GRC	-3.2324	5.2986	1.4290	ESP	-0.3413	0.8609	2.8447
GTM	-3.2324	5.2986	1.4290	SWE	-3.2324	5.2986	1.4290
HND	-3.2324	5.2986	1.4290	CHE	-8.3124	0.7343	1.4290
HUN	-3.2324	5.2986	0.3639	THA	-3.2928	5.2986	0.9913
IDN	-4.3363	5.2986	2.3386	ARE	-3.2324	5.2986	1.4290
IRL	-3.2324	0.1385	1.4290	TUR	-3.2324	5.2986	0.3238
ISR	-3.2324	5.2986	1.5406	UGA	-3.2324	5.2986	1.4290
ITA	-1.3687	1.4517	1.4290	UKR	-3.2324	5.2986	1.6535
JPN	-5.6492	9.2026	2.3297	MKD	-3.2324	5.2986	1.4290
KAZ	-1.9449	0.0000	4.0329	GBR	-3.2324	0.6962	2.1423
KEN	-3.2324	5.2986	1.4290	USA	-0.4143	7.1706	1.5247
KOR	-6.4940	2.9158	3.6755	URY	-3.2324	5.2986	1.4290
LVA	-3.2324	5.2986	1.4290	UZB	-3.2324	5.2986	1.4290
LTU	-3.2324	5.2986	1.4290	MNG	-3.2324	5.2986	1.4290
LUX	-3.2324	5.2986	1.4290	ROA	-3.2324	5.2986	0.5990
MDG	-3.2324	5.2986	1.4290				

Supplemental reference

[S1]. Wang, Z. Rare earth exports to be below quota. *China daily* (2012).

[S2]. Matthews, R. *et al.* The evolving landscape of trade controls on Critical Minerals and Rare Earth Elemen. *Dentons* (2025). Available at: https://www.dentons.com/en/insights/articles/2025/june/26/the-evolving-landscape-of-trade-controls-on-critical-minerals-and-rare-earth-elements.

[S3]. Zhang, H., Cao, H. & Guo, Y. The time-varying impact of geopolitical relations on rare earth trade networks: What is the role of China's rare earth export restrictions? *Technol. Forecast. Soc. Chang.* **206**, 123550 (2024).

[S4]. Philip, Andrews-Speed Anders, H. *China's rare earths dominance and policy responses*. (2023).

[S5]. Malik, J. A. N. China eliminates rare-earths quotas. *MRS Bull.* **40**, 206–207 (2015).

[S6]. Qiao, J. *et al.* The vital application of rare earth for future high-performance electromagnetic wave absorption materials: A review. *J. Mater. Sci. Technol.* **176**, 188–203 (2024).

[S7]. Ballinger, B. *et al.* The vulnerability of electric-vehicle and wind-turbine supply chains to the supply of rare-earth elements in a 2-degree scenario. *Sustain. Prod. Consum.* **22**, 68–76 (2020).

[S8]. Strategic Metals Prices. Rare Earth Element Prices. (2025). Available at: https://strategicmetalsinvest.com/current-strategic-metals-prices/.
